# One-Pot Synthesis of NiSe_2_ with Layered Structure for Nickel-Zinc Battery

**DOI:** 10.3390/molecules28031098

**Published:** 2023-01-21

**Authors:** Shi Chen, Yifeng Huang, Haoran Li, Fuxin Wang, Wei Xu, Dezhou Zheng, Xihong Lu

**Affiliations:** 1 School of Applied Physics and Materials, Wuyi University, Jiangmen 529020, China; 2 MOE of the Key Laboratory of Bioinorganic and Synthetic Chemistry, The Key Lab of Low-Carbon Chem & Energy Conservation of Guangdong Province, School of Chemistry, Sun Yat-sen University, Guangzhou 510275, China

**Keywords:** Ni-Zn battery, NiSe_2_, one-pot synthesis, cathode

## Abstract

Transition metal organic framework materials and their selenides are considered to be one of the most promising cathode materials for nickel-zinc (denoted as Ni-Zn) batteries due to their low cost, environmental friendliness, and controllable microstructure. Yet, their low capacity and poor cycling performance severely restricts their further development. Herein, we developed a simple one-pot hydrothermal process to directly synthesize NiSe_2_ (denotes as NiSe_2_-X based on the molar amount of SeO_2_ added) stacked layered sheets. Benefiting from the peculiar architectures, the fabricated NiSe_2_−1//Zn battery based on NiSe_2_ and the Zn plate exhibits a high specific capacity of 231.6 mAh g^−1^ at 1 A g^−1^, and excellent rate performance (162.8 mAh g^−1^ at 10 A g^−1^). In addition, the NiSe_2_//Zn battery also presents a satisfactory cycle life at the high current density of 8 A g^−1^ (almost no decay compared to the initial specific capacity after 1000 cycles). Additionally, the battery device also exhibits a satisfactory energy density of 343.2 Wh kg^−1^ and a peak power density of 11.7 kW kg^−1^. This work provides a simple attempt to design a high-performance layered cathode material for aqueous Ni-Zn batteries.

## 1. Introduction

With the continuous consumption of conventional energy resources and the increasing demand for energy, the energy crisis and the consequent environmental problems have attracted extensive attention. A large quantity of energy storage devices have been developed in the past few decades, such as supercapacitors, Li-ion batteries, and aqueous rechargeable batteries [1,2,3,4,5,6,7]. Among those aforementioned, energy storage devices such as aqueous Zn-based batteries are attracting considerable attention due to their high theoretical capacity (5851 mAh cm^−3^ and 820 mAh g^−1^), low redox potential (0.763 V vs. the standard hydrogen electrode (SHE)), high output voltage (such as Ni-Zn battery about 1.8 V), good compatibility with aqueous electrolytes, eco-friendliness, low cost, and abundant source of the Zn anode [8,9,10,11,12,13,14,15,16]. Compared to the high theoretical capacities of zinc anode materials, most reported cathode materials have relatively low capacities (200–300 mAh g^−1^) [1,5,17,18,19,20,21,22]. In this scenario, the key to improving the capacity of Ni-Zn battery is to explore suitable cathode materials.

Among current cathode materials, transition metal selenides (TMSs) own higher conductivity than transition metal oxides and hydroxides. For example, the binding energy of Ni-Se is lower than that of Ni-O and Ni-S with weaker electronegativity, reducing the energy consumption of conversion reaction [23]. Additionally, due to the higher degree of reversibility of M-Se compared to M-O bonds (M = metal), TMSs always exhibit superior electrochemical performance such as high specific capacity, rate capability, and long-term cyclability [24,25,26,27,28]. Due to the porous structure and easy adjustment, MOFs are ideal precursor materials for the construction of TMSs, which have been identified as cathode candidates for zinc-based batteries. The synthesized TMSs through the above method not only own controllable nanostructures but also show greater electrochemical performance [6,7,29,30,31,32]. For instance, CoSe_2-x_-derived cobalt oxides nanoparticles synthesized by Tang et al. obtained a capacity of 7.42 mAh m^−2^ at 10 mA cm^−2^ with 0.02% cycle^−1^ capacity decay over 4200 cycles [19]. Porous NiCoSe_2_@NiOOH/CoOOH nanoflakes reported by Cui and his co-workers exhibited a capacity of 108.9 mAh g^−1^ at 1 A g^−1^ with 105.1% capacity retention after 4000 cycles [33]. MoSe_2_ decorated Ni/Co selenide complex hollow arrays presented a capacity of 142 mAh g^−1^ at 2 A g^−1^ with ~36% capacity loss after 1000 cycles [34]. However, the capacity and long cycle stability of most reported TMSs//Zn batteries have still fallen below expectations and cannot meet future practical applications. Moreover, the traditional method of constructing selenide by MOFs is relatively complicated, which requires high temperature carbonization followed by high-temperature selenization under Ar flow. Therefore, the investigation of new durable TMSs cathodes with high capacity is still highly favorable and challenging for achieving widely applicable aqueous rechargeable zinc-based batteries.

In this work, we propose a facile one-pot hydrothermal synthesis of NiSe_2_ stacked layered sheets without Ni-MOFs template and high temperature heat treatment. The NiSe_2_//Zn battery was assembled by using the synthesized NiSe_2_ nanosheet as the cathode and zinc as the anode of the aqueous Ni-Zn batteries, and the fabricated battery obtained a higher specific capacity of 231.6–162.8 mAh g^−1^ at various current densities of 1–10 A g^−1^, which indicated the good rate performance. Moreover, it shows excellent cycling performance with almost no decay compared to the initial specific capacity value after 1000 cycles at 8 A g^−1^. Impressively, the fabricated NiSe_2_//Zn battery shows outstanding performance with a maximum energy density of 343.2 Wh kg^−1^, and a maximum power density of 11.7 kW kg^−1^, substantially outstripping most of the recently reported Ni-Zn batteries.

## 2. Results and Discussions

**Figure 1:**
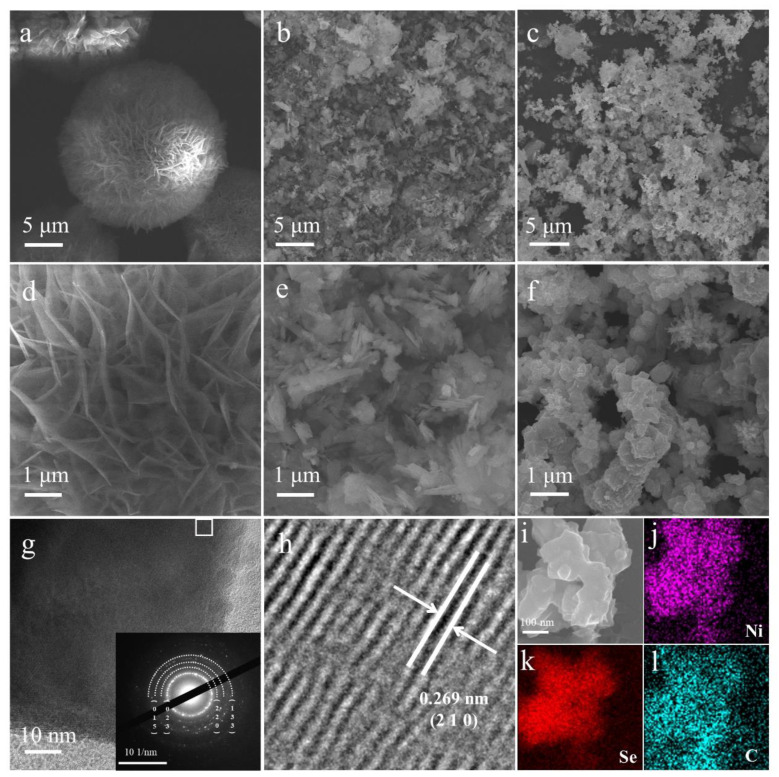
SEM images of the (**a**) Ni-MOFs, (**b**) NiSe_2_−0.5, and (**c**) NiSe_2_−1 samples at low magnification. The SEM images of the corresponding area of (**d**) Ni-MOFs, (**e**) NiSe_2_−0.5, and (**f**) NiSe_2_−1 samples at high magnification. (**g**) TEM image of NiSe_2_−1 sample and corresponding SAED pattern (inset). (**h**) HRTEM image of NiSe_2_−1 sample. (**i**–**l**) EDS mapping.

The Ni-MOFs nanomaterials were first synthesized by a simple one-pot hydrothermal process. As shown in Figure 1a, the pristine Ni-MOFs nanomaterials own a cluster structure stacked with numerous layered sheets, and the self-assembled nanosheets can be better observed in the high magnification SEM images (Figure 1d). Based on the above synthesis process, NiSe_2_−X nanomaterials with different degrees of selenization can be obtained by adding different masses of SeO_2_ into the precursor solution of Ni-MOFs synthesis. Figure 1b–c and Figure S1 show the variation of the structure of NiSe_2_ nanosheets under different SeO_2_ addition conditions. It can be seen that NiSe_2_−0.5 still retains part of the original nanosheets due to the lack of SeO_2_ during the synthesis process. Meanwhile, in Figure 1f, the homogeneous growth and accumulation of thick nanosheets of NiSe_2_−1 can be observed. Through the high magnification SEM images of the corresponding area (Figure 1e, f and Figure S1b), it can be clearly observed that the thickness of nanosheets increased with the increased concentration of SeO_2_. Figure 1i–l shows the EDS images of NiSe_2_−1 nanomaterials, which indicates that Ni, Se, and C elements are uniformly distributed in the sample. In order to further explore the microstructure of Ni-MOFs and NiSe_2_-1 nanomaterials, we utilized TEM (Figure 1g and Figure S2). Figure 1g shows that the NiSe_2_-1 nanomaterials consisted of the square-shaped nanosheets. The inset picture in Figure 1g shows the corresponding selected area electron diffraction (SAED) pattern indicating the typical polycrystalline structure of NiSe_2_−1. The lattice fringes of the NiSe_2_−1 material were visible in Figure 1h, where the characteristic lattice spacing (0.269 nm) of NiSe_2_−1 was consistent with the (210) lattice plane of NiSe_2_ (JCPDS NO. 41-1495) [35].

**Figure 2:**
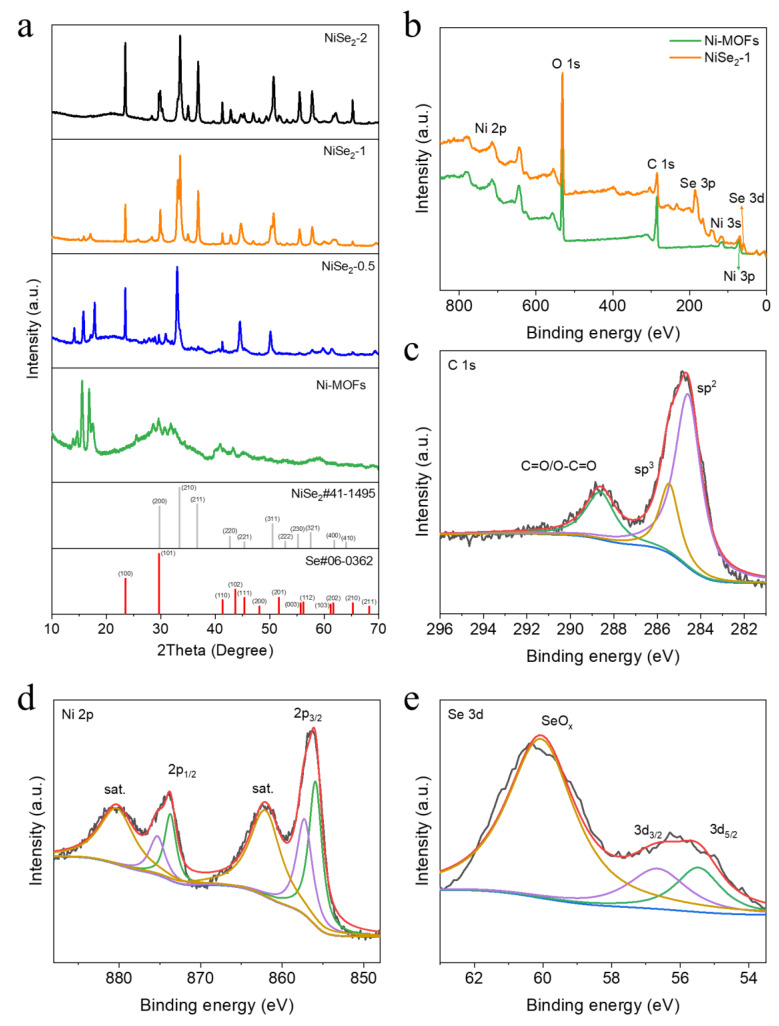
(**a**) XRD patterns of Ni-MOFs and NiSe_2_−X (X = 0.5, 1, 2). (**b**) XPS survey of the Ni-MOFs and NiSe_2_−1 samples. (**c**) High resolution XPS spectra of C 1s. (**d**) Ni 2p and (**e**) Se 3d XPS spectra.

Figure 2a shows the XRD spectra of the Ni-MOFs and NiSe_2_−X samples, which obviously demonstrates that the characteristic peaks of the four samples have changed significantly due to the different amount of SeO_2_ added during the synthesis process. The XRD diffraction patterns of NiSe_2_−1 sample, displaying six peaks at 2θ = 29.8°, 33.4°, 36.7°, 50.5°, 55.2° and 57.5° respectively, which are a right match (200), (210), (211), (311), (230), and (321) planes of NiSe_2_ (JCPDS NO. 41-1495), were also related to the lattice fringes of the NiSe_2_−1 material (Figure 1h). Additionally, the characteristic peaks of NiSe_2_−0.5 sample contain part characteristic peaks of Ni-MOFs, indicating incomplete transformation, which is consistent with the SEM results. To further explore the elemental composition and valence state of the samples, XPS was used to analyze the as-synthesized samples. Figure 2b is the survey spectra of Ni-MOFs and NiSe_2_−1 samples. It can be clearly seen that the NiSe_2_−1 sample synthesized by adding SeO_2_ has an obvious characteristic peak of Se, indicating the successful doping of Se. The C 1s spectrum of NiSe_2_−1 sample can be resolved into three characteristic peaks of sp^2^-C, sp^3^-C, C=O, and O-C=O bonds, respectively (Figure 2c) [22,23]. From the Ni 2p spectrum (Figure 2d), two peaks located at 855.9 and 873.7 eV were attributed to Ni^2+^ in Ni 2p^3/2^ and Ni 2p_1/2_ respectively. Meanwhile, the peaks located at 857.3 and 875.3 eV corresponded to the Ni^3+^ in Ni 2p_3/2_ and Ni 2p_1/2_, and the others were related to the satellite peaks [22,36]. As displayed in Figure 2e, the Se 3d_5/2_, 3d_3/2_, and SeO_x_ level appeared at 55.7, 56.9 and 60.2 eV, respectively [35,37,38]. Additionally, the spectra of O 1s for Ni-MOFs and NiSe_2_−1 samples can be deconvoluted into three peaks assigned as Se-O (530.8 eV), OH^-^ (533.6 eV) and adsorb water (532.5 eV), respectively (Figure S3) [35].

**Figure 3:**
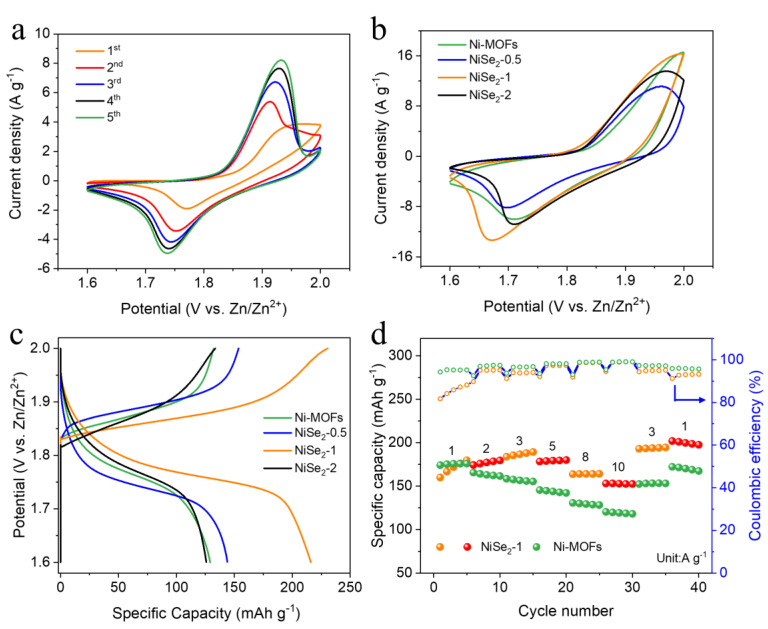
(**a**) CV curves of NiSe_2_-1 at a scan rate of 1 mV s^−1^ for initial preactivation. (**b**) CV curves of Ni-MOFs and NiSe_2_−X (X = 0.5, 1, 2) at the same scan rate of 5 mV s^−1^. (**c**) GCD curves of Ni-MOFs and NiSe_2_−X (X = 0.5, 1, 2) at the same current density of 3 A g^−1^. (**d**) Rate performances and CE of Ni-MOFs and NiSe_2_−1.

In order to evaluate the electrochemical performance of the prepared electrode materials, we assembled Ni-MOFs, NiSe_2_−X and Zinc plate into aqueous Ni-Zn batteries. Figure 3a shows different cyclic voltammetry (CV) curves of NiSe_2_-1 in the initial five cycles. It can be clearly observed that the current response signal increases with the number of scans, indicating the improvement of its electrochemical performance. This phenomenon can be attributed to the continuous activation of the electrode as a result of the surface chemical reaction between NiSe_2_−1 and KOH electrolyte [39,40]. Figure 3b shows the CV curves of the fabricated batteries with the working window from 1.6-2.0 V, which exhibit typical battery behavior with obvious redox peaks. As illustrated in Figure 3c, the NiSe_2_−1 displays the highest specific capacity, while the GCD curves of the as-prepared Ni-MOFs and NiSe_2_−X samples are at the same current density (3 A g^−1^). The corresponding GCD curves of NiSe_2_-1 at different current densities are depicted in Figure S4, and it can be clearly seen that the discharge specific capacity reached a remarkable 231.6 mAh g^−1^ at 1 A g^−1^, and the discharge specific capacity was still maintained at 162.8 mAh g^−1^ with a high current density of 10 A g^−1^, proving its excellent rate performance. Meanwhile, the obvious charge/discharge voltage plateaus appear in the GCD curves at different current densities, and the voltage range of the plateau (at 1.85–1.95 V) matches the position of the redox peaks in the cyclic voltammetry curves correspondingly. This result further confirms that the NiSe_2_-1 electrode undergoes a redox reaction, which has the typical characteristics of a battery-type material. The rate performance of the original Ni-MOFs and NiSe_2_−1 electrodes applied to Ni-Zn batteries is shown in Figure 3d. It is noteworthy that the NiSe_2_−1 exhibits an apparent increase in specific capacity during the initial cycles, which follows the same trend as that in Figure 3a. Both rate capability curves can illustrate that there is no significant decay after the rate cycling due to the good rate capability of Ni-based materials [23]. However, when the current density falls back to 1 A g^−1^ after 40 cycles, the average capacity retention of the NiSe_2_−1 electrode can even reach 117% compared to the initial average capacity, which is better than that of the Ni-MOFs electrode (96%) [41].

**Figure 4:**
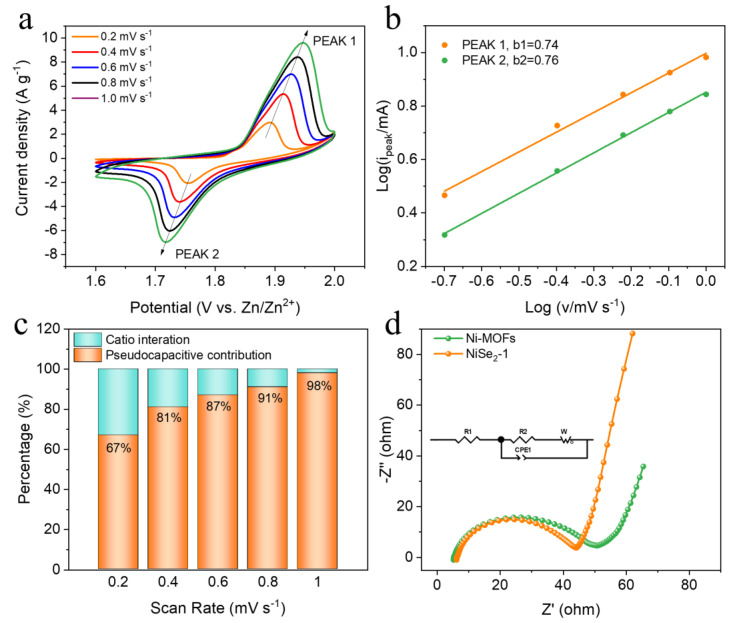
(**a**) CV scans of NiSe_2_−1 at various rates. (**b**) Correspondingly fitted *b*-value at respective redox peaks. (**c**) Relative contribution of the capacitive and diffusion-controlled charge storage at different scan rates. (**d**) Nyquist plots of Ni-MOFs and NiSe_2_−1.

In order to further understand the storage mechanism of Zn^2+^, we used different electrochemical methods to explore its kinetics. Figure 4a shows the cyclic voltammetry (CV) curves of NiSe_2_−1 electrode at different scan rates. It is obvious that with the increase of the scanning rate, the peak pattern of the redox peaks does not change, but the position of the redox shifts, which is mainly attributed to polarization. Theoretically, the peak current (*i*) and the scan rate (*v*) of the CV curves follow the equation: *i* = *av^b^*, where *a* and *b* are variable parameters. A *b* value of 0.5 indicates a diffusion-controlled behavior of the Zn-ion extract/insert electrode material, and a *b* value of 1 indicates a control by the capacitive process. The *b* values can be obtained by fitting a linear relationship between *log i* and *log v* [42,43]. As exhibited in Figure 4b, the *b* values at each redox peak of the NiSe_2_−1 electrode are 0.74 and 0.76, respectively, between 0.5 and 1, suggesting the presence of both diffusion-controlled and capacity-based kinetic processes. The pseudocapacitive contributions of NiSe_2_−1 at 0.2, 0.4, 0.6, 0.8, 1 mV s^−1^, presented in Figure 4c, based on the pseudocapacity calculation equation *i* = *k_1_v* + *k_2_v^1/2^*, where *k_1_* and *k_2_* are constant values, and the calculated result of it is the theoretical value of the pseudocapacitive contribution [44,45]. For the as-prepared NiSe_2_-1 electrode, the *k_1_v* value ranges from 67% to 98% at the scan range of 0.2–1 mV s^−1^. Typically, the original CV curve and fitted pseudocapacitive contribution (the shaded area) at 1 mV s^−1^ are shown in Figure S5. In addition, the capacity contribution still dominates at the lowest scan rate. Under the influence of pseudocapacitance behavior, the active material NiSe_2_−1 tends to undergo redox reaction on the electrode surface to store charge, which can effectively protect the structure of the NiSe_2_−1 and improve the cyclic stability of the electrode material. More importantly, the near-surface pseudocapacitance effect can obtain a shorter ion transport path and a faster electron transport rate, which is conducive to enhancing the electrochemical performance of NiSe_2_−1 [23]. The electrochemical impedance spectroscopy (EIS) in Figure 4d, where the equivalent circuit for simulation was inset, shows that the Nyquist plot consists of the semicircle in the high-frequency range related to the charge-transfer resistance and the low-frequency regions related to a diffusion-limited electron transfer process of an electrode [46,47]. It can be clearly seen that with the addition of SeO_2_ (Figure S6), the structure of the material gradually becomes thicker and reacts completely. The corresponding EIS of Ni-MOFs and NiSe_2_−X (X = 0.5, 1, 2) are depicted in Figure S7. Apparently, in the high frequency region, the R_ct_ of NiSe_2_-1 electrode is 34.31 Ω, while the R_ct_ of Ni-MOFs electrode is 40.6 Ω; in the low frequency region, NiSe_2_-1 electrode has a higher slope of the line than Ni-MOFs electrode, which means a faster electron diffusion. Therefore, the NiSe_2_−1 electrode exhibits better impedance performance than the pristine Ni-MOFs in both low and high frequency regions.

**Figure 5:**
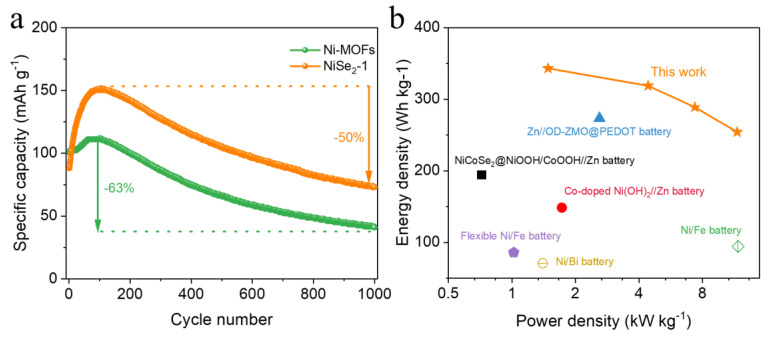
(**a**) Long-term cycling stability of Ni-MOFs and NiSe_2_−1 at 8 A g^−1^ over 1000 cycles. (**b**) Ragone plot of the NiSe_2_−1//Zn battery and other reported rechargeable batteries [9,21,33,48,49,50].

In order to further explore the practicability of the Ni-Zn batteries prepared, we investigated the cycle performance, energy density, and power density. Figure 5a displays the comparison of the long cycle performance of Ni-MOFs and NiSe_2_−1 as the cathode electrode of Ni-Zn batteries. It can be seen that the specific capacity of Ni-MOFs is slightly higher than that of NiSe_2_−1 during the very initial cycles, corresponding to the initial rate capability curve in Figure 3e. However, it is worth noting that the specific capacity of NiSe_2_−1 has a rather high percentage increase compared to the pristine Ni-MOFs, and the increase in capacity over about 100 cycles is attributed to the surface chemistry between NiSe_2_−1 and the KOH electrolyte, which activates the electrode [39,40]. After reaching the peak specific capacity value, both materials began to decay slowly, and after 1000 cycles, NiSe_2_-1 decayed by 50% of the peak capacity (Ni-MOFs, 61%), as shown within Figure 5a. Additionally, based on the GCD curves, the energy density and power density of the NiSe_2_−1//Zn battery were calculated to obtain the Ragone plots (Figure 5b). Noteworthily, the NiSe_2_−1//Zn battery obtained a maximum energy density of 343.2 Wh kg^−1^ and a peak power density of 11.7 kW kg^−1^, which is superior to most of the previously reported rechargeable batteries, including Ni//Bi battery (1.4 kW kg^−1^, 70.9 Wh kg^−1^) [9], Zn//OD-ZMO@PEDOT battery (273.4 Wh kg^−1^, 2.6 kW kg^−1^) [21], NiCoSe_2_@NiOOH/CoOOH//Zn battery (0.72 kW kg^−1^, 194.2 Wh kg^−1^) [33], Co-doped Ni(OH)_2_//Zn battery (1.725 kW kg^−1^, 148.54 Wh kg^−1^) [48], Ni//Fe battery (11.8 kW kg^−1^, 94.5 Wh kg^−1^) [49] and flexible Ni/Fe battery (1.02 kW kg^−1^, 85.8 Wh kg^−1^) [50].

## 3. Experimental Section

### 3.1. Synthesis of Ni-MOFs

All chemicals in this work were utilized as received without any purification (the purity of chemicals was 98%). 1 mmol Ni(NO_3_)_2_·6H_2_O and 1 mmol PTA were dissolved in a mixed solution consisting of 20 mL of DMF and 5 mL of 0.4 M NaOH, stirred well at room temperature and transferred into a 50 mL Teflon-lined stainless steel reactor, which was subjected to hydrothermal reaction at 160 °C for 12 h in an oven. After the reaction, the solution was cooled down to room temperature, centrifugally, and dried at 60 °C for 12 h to obtain Ni-MOFs.

### 3.2. Synthesis of NiSe_2_ Architectures

The synthesis of NiSe_2_ is based on the above synthesis method. It only needs to add different masses of SeO_2_ to the precursor solution for the synthesis of Ni-MOFs. The resulting products were named as NiSe_2_−X (X = 0.5,1,2) according to the molar amount of SeO_2_ added.

### 3.3. Fabrication of Aqueous Ni-Zn Batteries and Electrochemical Measurements

The aqueous Ni-Zn batteries were fabricated by a piece of Zn plate, the synthesized NiSe_2_−X (X = 0.5, 1, 2) electrode, Zn flakes, and a mixed aqueous consisting of 3 M KOH and 0.2 M Zn(CH_3_COO)_2_ as the anode, cathode, and electrolyte. The mass loading was about 2.4 mg cm^−2^. The electrochemical performances (CV, CP, EIS) of the fabricated batteries were characterized by an electrochemical workstation (CHI 660E) and Land 2001A.

### 3.4. Materials Characterization

The surface morphology, microstructure, crystalline structure, chemical composition, and elemental valence of the synthesized samples were investigated by the field-emission scanning electron microscope (SEM, Sigma500, ZEISS, Oberkochen, Germany) equipped with an energy dispersive X-ray element analysis system (EDS), transmission electron microscope (TEM, JEM-F200, JEOL, Tokyo, Japan), X-ray diffraction (XRD, X ’Pert Pro MPD, PANalytical, Malvern, UK) patterns, X-ray photoelectron spectroscopy (XPS, NEXSA, Thermo VG, Waltham, MA, USA), and Raman spectroscopy (LabRAM HR Evolution, HORIBA, Kyoto, Japan).

All electrochemical characterization was carried out in a two-electrode system with a potential window of 1.6 to 2.0 V. Galvanostatic charge/discharge (GCD), Cyclic voltammetry (CV) and electrochemical impedance spectroscopy (EIS) measurements were performed using an electrochemical workstation (CHI 660E). The cycle life of the Ni-Zn batteries was tested by using a Land 2001A.

## 4. Conclusions

In summary, based on the synthesis of Ni-MOF, we have developed a simple one-pot synthesis method of NiSe_2_ with a layered structure. Benefiting from the unique nanosheet structure, the assembled aqueous NiSe_2_−1//Zn battery exhibits remarkable specific capacity (231.6 mAh g^−1^ at 1 A g^−1^), excellent rate performance (162.8 mAh g^−1^ at 10 A g^−1^), and prominent energy density (343.2 Wh kg^−1^) as well as excellent long-term cycling stability (almost no decay after 1000 cycles at 8 A g^−1^ compared to the initial specific capacity value). Moreover, NiSe_2_ can be easily prepared by a one-pot hydrothermal synthesis method, which facilitates the widespread and commercial application of the energy storage devices and lays the foundation for future research and development of such materials.

## Data Availability

Data available in a publicly accessible repository.

## Supplementary Materials

The following supporting information can be downloaded at: https://www.mdpi.com/article/10.3390/molecules28031098/s1, Figure S1: SEM images of the NiSe_2_−2 sample (a) at low magnification, (b) at high magnification; Figure S2: TEM image of (a) Ni-MOFs sample. (b) NiSe_2_−1 sample; Figure S3: High resolution O 1s XPS spectra of (a) Ni-MOFs and (b) NiSe_2_−1; Figure S4: GCD curves of NiSe_2_−1 at different current densities; Figure S5: Ratio of pseudo-capacitive capacity for NiSe_2_−1 at 1 mV s^−1^; Figure S6: SEM images of (a) Ni-MOFs, (b) NiSe_2_−0.5, (c) NiSe_2_−1 and (d) NiSe_2_−2 samples; Figure S7: Nyquist plots of Ni-MOFs and NiSe_2_−X (X = 0.5, 1, 2).

## Sample Availability

Samples of the compounds are not available from the authors.
