# Supplementary material for: One-Pot Synthesis of NiSe2 with Layered Structure for Nickel-Zinc Battery
Source: Molecules. 2023 Jan 21;28(3):1098. doi: 10.3390/molecules28031098 (PMC9919136; doi:10.3390/molecules28031098)
Supplement: Supplementary file 1 [file molecules-28-01098-s001.zip › molecules-2154640-supplementary.pdf]

One-pot Synthesis of $NiSe_2$ with layered Structure for Nickel-Zinc battery

Shi Chen [1], Yifeng Huang [1], Haoran Li [1], Fuxin Wang [1,*], Wei Xu [1], Dezhou Zheng [1], Xihong Lu [1,2,*]

[1] *School of Applied Physics and Materials, Wuyi University, Jiangmen 529020, PR China*

[2] *MOE of the Key Laboratory of Bioinorganic and Synthetic Chemistry, The Key Lab of Low-carbon Chem & Energy Conservation of Guangdong Province, School of Chemistry, Sun Yat-Sen University, Guangzhou 510275, PR China*

* *Correspondence: wangfux91@126.com; luxh6@mail.sysu.edu.cn.*

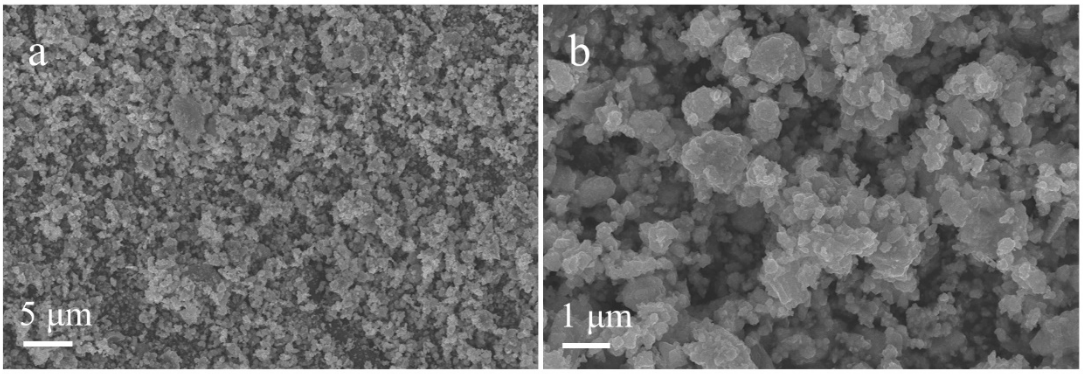

Figure S1 SEM images of the $NiSe_2$-2 sample (a) at low magnification, (b) at high magnification.

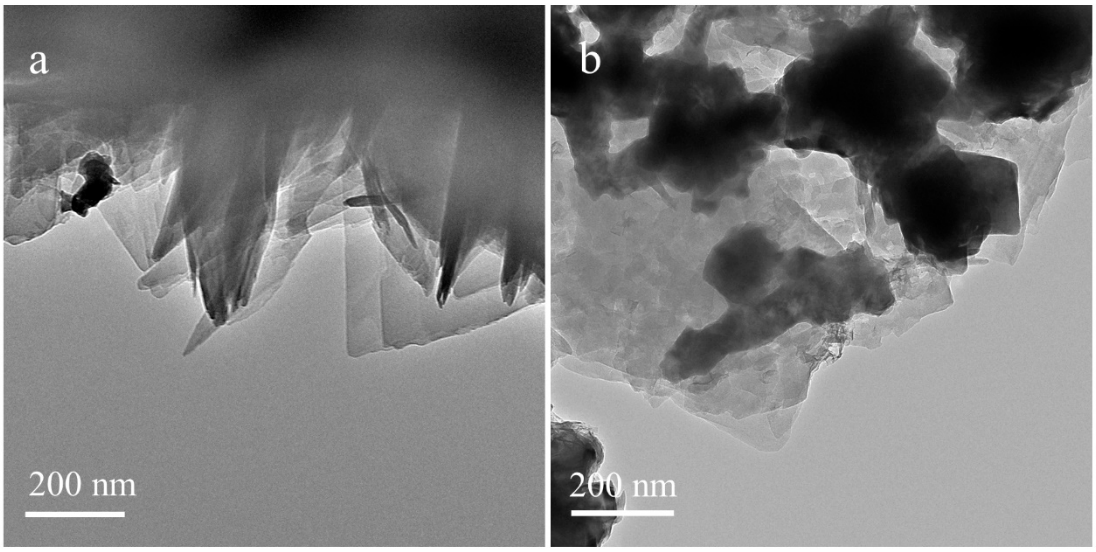

Figure S2 TEM image of (a) Ni-MOFs sample and (b) $NiSe_2$-1 sample.

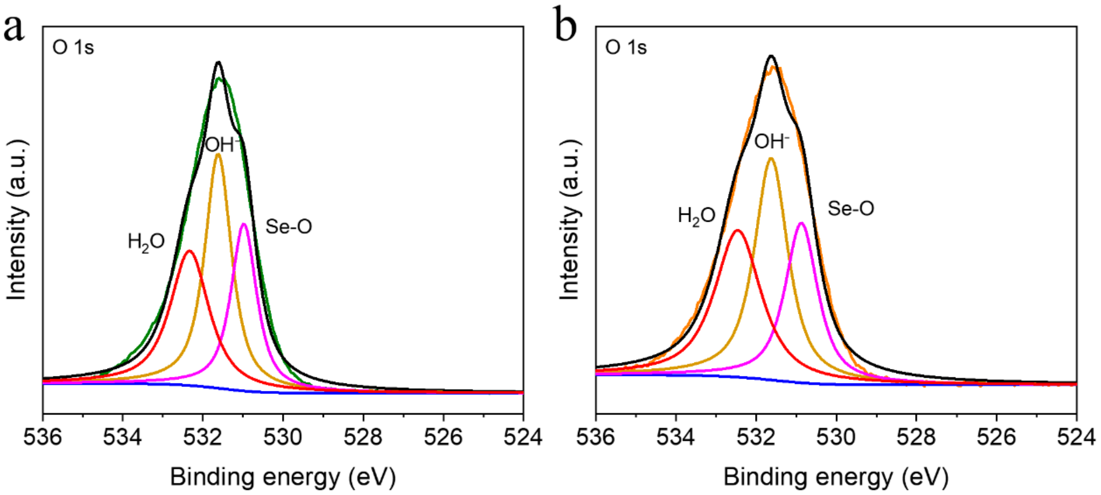

Figure S3 High resolution O 1s XPS spectra of (a) Ni-MOFs and (b) $NiSe_2$-1.

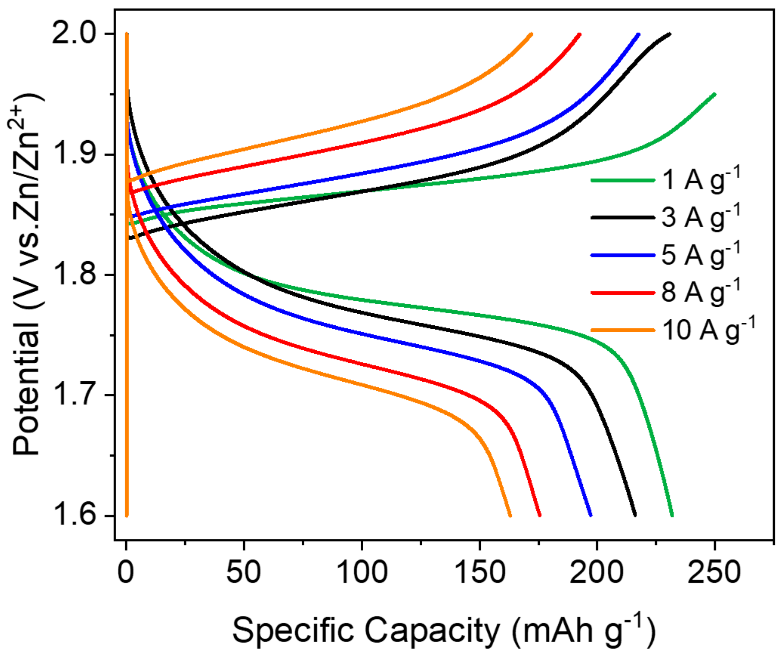

Figure S4 GCD curves of $NiSe_2$-1 at different current densities.

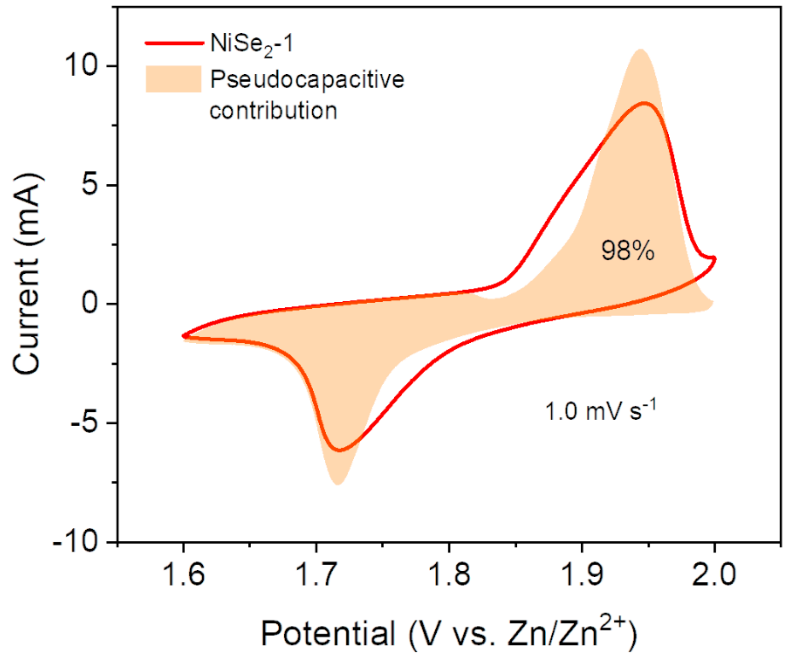

Figure S5 Ratio of pseudo-capacitive capacity for $NiSe_2$-1 at 1 mV s^{-1}.

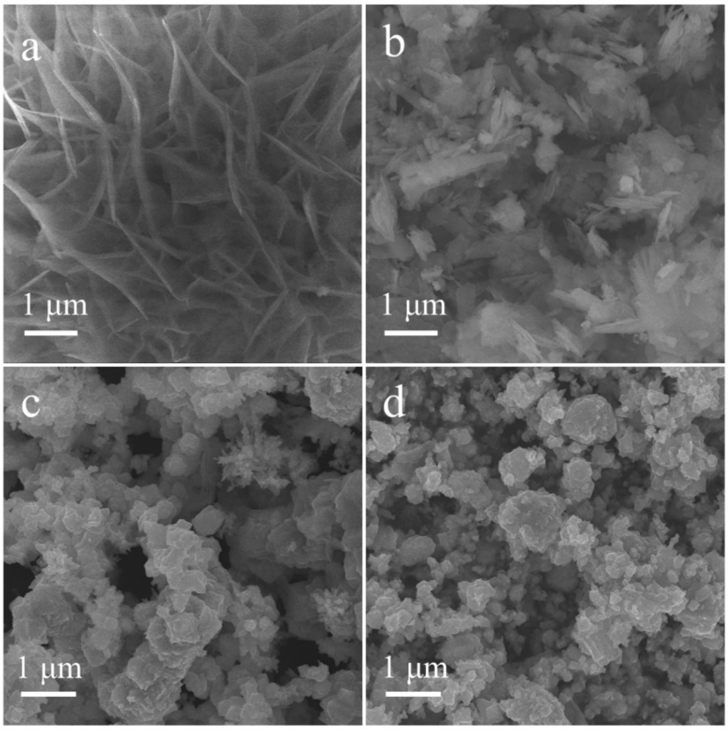

Figure S6 SEM images of (a) Ni-MOFs, (b) $NiSe_2$-0.5, (c) $NiSe_2$-1 and (d) $NiSe_2$-2 samples.

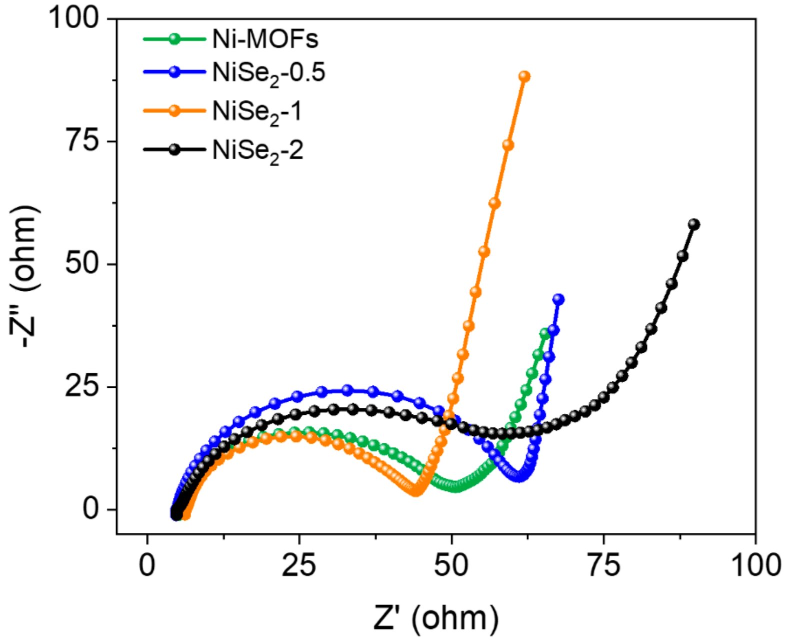

Figure S7 Nyquist plots of Ni-MOFs and $NiSe_2$-X (X=0.5,1,2).
